# Diagnostic moléculaire du complexe *Mycobacterium tuberculosis* résistant à l'isoniazide et à la rifampicine au Burkina Faso

**DOI:** 10.11604/pamj.2015.21.73.5494

**Published:** 2015-05-29

**Authors:** Ilboudo Désire, Bisseye Cyrille, Djigma Florencia, Diande Souba, Yonli Albert, Bazie Jean Telesphore Valerie, Compaore Rebecca, Gnoula Charlemagne, Djibril Tamboura, Moret Rémy, Pietra Virginio, Karou Damintoti Simplice, Ouedraogo Martial, Simpore Jacques

**Affiliations:** 1 Centre de Recherche Biomoléculaire Pietro Annigoni, CERBA/LABIOGENE, Université de Ouagadougou, Ouagadougou, Burkina Faso; 2 Laboratoire National de Référence de Mycobactéries, Programme National Tuberculose, Ouagadougou, Burkina Faso; 3 Centre Médical St Camille, Ouagadougou, Burkina Faso; 4 Ecole Supérieure des Techniques Biologiques et Alimentaires (ESTBA-UL), Université de Lomé, Lomé, Togo; 5 Unité de Formation et de Recherche en Sciences de la Santé (UFR/SDD), Université de Ouagadougou, Ouagadougou, Burkina Faso

**Keywords:** Mycobacterium tuberculosis, résistance, rifampicine, isoniazide, Mycobacterium tuberculosis, resistance, rifampicin, isoniazid

## Abstract

**Introduction:**

Cette étude a eu pour objectifs de diagnostiquer la tuberculose pulmonaire par l'examen microscopique et par la PCR des crachats et de déterminer les bases moléculaires de la résistance à la rifampicine et à l'isoniazide.

**Méthodes:**

Le diagnostic du Complexe *Mycobacterium Tuberculosis* (CMTB) a été effectué par microscopie après coloration au Ziehl Nielsen et par PCR en temps réel en utilisant le kit d'identification du complexe MTB (Sacace Biotechnologie, Italie). Les résistances à la Rifampicine et à l'Isoniazide ont été étudiées par la technique de la PCR en utilisant le kit MTB résistance 8 (Sacace, Biotechnologie).

**Résultats:**

Sur les 59 patients diagnostiqués pour la tuberculose pulmonaire, 59,3% étaient positifs en microscopie optique et 44,1% étaient positifs par PCR en Temps réel. Les résistances à la rifampicine (*rpo*B) et à l'isoniazide (k*at*G et *inh*A) ont été observées chez 9 patients. La résistance à la rifampicine était due aux mutations (Asp516Val, Ser531Trp, Leu533Pro) et celle à l'isoniazide par les substitutions Ser315Thr du gène *kat*G et C209T du gène *inh*A. Les multi résistances à la rifampicine et à l'isoniazide ont été observées dans 55,5% des échantillons et concernaient les associations: *rop*BAsp513Val + *inh*AC209T et *rpo*BLeu533Pro + *kat*GSer315Thr.

**Conclusion:**

La PCR en temps réel qui permet l'identification des allèles mutants *rpo*B, katG et *inh*A de *M. tuberculosis* est un outil de diagnostic épidémiologique de grande importance car elle permet de déterminer le niveau de résistance à la rifampicine et à l'isoniazide.

## Introduction

L'augmentation des cas de tuberculoses pharmaco-résistantes constitue un problème de santé publique au niveau mondial. Selon des données de l'OMS, en 2011, environ 12 millions de cas de tuberculose ont été recensés dans le monde dont plus de 630 000 cas de tuberculose multi-résistante (TB-MR) [1]. Ces tuberculoses multi-résistantes sont des cas où les bacilles de Koch résistent non seulement à l'isoniazide mais aussi à la rifampicine qui sont les deux principaux médicaments antituberculeux. Au niveau mondial, le taux de succès des traitements des nouveaux cas de tuberculoses pulmonaires à frottis positif était de 87% en 2009 et de 76% au Burkina Faso à la même période [2, 3]. En 1997 en Côte d'Ivoire au total 192 cas de tuberculose à microscopie positive ont été enregistrés au CHU de Treichville dont 8 ont été confirmés résistants à la rifampicine et à l'isoniazide [4], de même au Mali en 2010, le taux de TB MR avait atteint 35,7% de cas sur 126 cas suspects [5].

Au Burkina Faso, la plus part des cas notifiés sont issus des études dans les principaux services de pneumologie du pays. En 2009, dans une étude portant sur la détection moléculaire de la résistance à la rifampicine et à l'isoniazide, sur les 108 cas chroniques de tuberculose enregistrés, 24 ont été confirmés résistants à la rifampicine et à l'isoniazide [6]. Entre 2006 et 2009, au total 156 patients atteints de tuberculose chronique ont été enregistrés au Burkina Faso dont 50 échantillons ont été révélés positifs, par culture, à *Mycobacterium tuberculosis*. Sur ces 50 mycobactéries identifiés, 34 (68%) ont été confirmés muti-résistants et deux cas de tuberculose ultra résistante (XR), à savoir, des mycobactéries résistantes à la rifampicine, à l'isoniazide et à d'autres médicaments antituberculeux [7]. En 2010 également, l’étude de Sangaré et al. sur les résistances aux médicaments anti-mycobactéries chez les nouveaux cas de tuberculose pulmonaire ou traités antérieurement au Burkina Faso, a révélé une haute résistance pour l'isoniazide (66,7%) et à la rifampicine (51,6%) avec un taux de TB-MR de 50,5% des cas [8].

Le diagnostic des TB-MDR est apporté par la mise en évidence des mycobactéries, par culture suivie de l'antibiogramme, ou par la détection de la présence d'ADN ou d'ARN mycobactérien muté, conférant une résistance à un antibiotique donné. La détermination rapide de la présence du génome bactérien par PCR constitue un progrès récent dans le diagnostic de la tuberculose. Dans les cas graves, où l’établissement rapide d'un diagnostic et la mise immédiate sous traitement antituberculeux sont impératifs, la PCR permet de gagner du temps en révélant la présence d'antigènes mycobactériens alors que l'examen microscopique direct est négatif et que la preuve de la présence de mycobactéries viables sera apportée deux à huit semaines plus tard par culture [9]. Au Burkina Faso, les tests de résistance aux antituberculeux ne sont pas réalisés systématiquement. Après un dépistage microscopique, les patients suspects d'avoir une tuberculose sont d'abord traités suivant un schéma standard de l'OMS qui préconise des thérapies de 3 à 4 antibiotiques (Rifampicine, Isoniazide, Pyrazynamide et Ethambutol) pendant deux mois, suivi de la prise de Rifampicine et Isoniazide pendant 4 mois [10]. C'est après échec du traitement que la recherche de résistance est faite par culture. Pour le diagnostic rapide des TB-MR par PCR, les crachats sont envoyés en Europe, alors que la prise en charge de ces patients-MR est une urgence médicale. Cette étude a eu pour objectifs: i) d'identifier le complexe *Mycobacterium tuberculosis* par microscopie et par PCR en temps réel à partir des expectorations, ii) de caractériser le profil génétique de résistance du complexe *Mycobacterium tuberculosis* à la rifampicine et à l'isoniazide par PCR en temps réel et enfin, iii) de déterminer le profil de résistance simultanée du complexe *Mycobacterium tuberculosis* résistant à la rifampicine et l´isoniazide.

## Méthodes

### Cadre de l’étude

Cette étude a eu pour cadres l'hôpital de district de Bogodogo à Ouagadougou, le Centre Médical Saint Camille, le Programme National Tuberculose du Burkina Faso pour la collecte des échantillons et le Centre de Recherche Biomoléculaire Pietro Annigoni (CERBA/LABIOGENE) pour l'analyse des échantillons. Les patients enrôlés dans cette étude étaient au nombre de 74 avec une moyenne d’âge de 38,52 ± 14,02 ans. Ils étaient venus au laboratoire soit pour un dépistage de la tuberculose, soit pour un contrôle pendant ou après traitement de première ligne.

### Prélèvements

Les prélèvements ont été essentiellement des produits d´expectoration spontanée ou provoquée de 3 à 5 ml recueillis dans des pots stériles. Deux échantillons de crachat par malade ont été utilisés pour le dépistage et un échantillon pour chaque patient venu pour le contrôle sous traitement. D'autres souches de Mycobactéries déjà identifiées ont été utilisées pour l'identification du complexe de Mycobacterium et l’étude des résistances.

#### Examen microscopique

Sur chaque échantillon, un frottis a été confectionné et coloré par la technique de Zielh Neelsen à chaud. Les lames ont été lues au microscope optique et les BAAR ont été comptés sur 20 à 300 champs microscopiques selon la richesse du frottis.

#### Identification de Mycobacterium tuberculosis par PCR en temps réel

*Mycobacterium tuberculosis* a été identifié par PCR en temps réel à partir des crachats de patients grâce au kit d'identification MTB Real-TM (Sacace Biotechnologies, Como, Italie). Ce kit permet la détection qualitative du complexe *Mycobacterium tuberculosis (M. tuberculosis, M. africanum, M. bovis, M. bovis BCG, M. microti)* dans les crachats, l´urine, les lavages bronchiques, les tissus et autres produits biologiques.

#### Extraction de l'ADN

Les échantillons ont été traités avec de la soude (NaOH 4%) à égal volume puis centrifugés à 3000 rpm pendant 15mn. Le surnagent a été éliminé puis le culot conservé pour l'extraction. L'extraction a été faite en utilisant le kit Magno-Sorb-Tub de Sacace Biothechnologies (Sacace, REF K505, Italy) en utilisant le protocole fourni par le fabricant.

#### Identification du complexe Mycobacterium tuberculosis et interprétation des résultats

La PCR en temps réel s'est effectuée avec l´appareil Applied Biosystems^®^ 7500 Fast Real Time PCR Systems en utilisant le kit d'identification MTB Real-TM (Sacace Biotechnologies, Como, Italie). Ce kit a permis la détection qualitative du complexe *Mycobacterium tuberculosis (M. tuberculosis, M. africanum, M. bovis, M. bovis BCG, M. microti)*. La Real time PCR s'est réalisée pour 40 cycles dans les conditions suivantes: 95 °C pour 15mn, 65 °C pour 30 s, 72 °C pour 15 s. Les résultats ont été interprétés par la présence de croisement de la courbe de fluorescence avec la ligne de seuil. *Mycobacterium tuberculosis* a été détecté sur canal FAM (vert) et l'IC DNA sur le canal JOE(Jaune)/HEX/Cy3.

#### Détection des gènes de résistances à la rifampicine et à l'isoniazide

La détection des mutations responsables des résistances à la rifampicine et l'isoniazide a été faite par PCR en temps réel en utilisant le kit commercial (MTB Real-TM Resistance 8 kit, Sacace Biotechnologies, Como, Italie) en suivant les instructions du fabricant.

## Résultats

### Population d’étude

L’étude a porté sur 74 échantillons comprenant 67 prélèvements de crachat et 7 souches de culture du complexe MTB. Parmi les 67 échantillons, 52 patients venaient d’être dépistés et n'avaient pas encore reçu de traitement antituberculeux et 15 patients sous traitement ont été reçus pour contrôle. Le Tableau 1 montre la répartition des sujets en fonction de leur sexe, groupe d’âge et de leur statut sérologique au VIH-1. La majorité des cas de tuberculoses a été retrouvée chez les patients de la tranche d’âge 21 à 30 ans (29,9%) et de 31 à 40 ans (28,4%). L´âge moyen des patients était de 38,52±14,02 ans. Les enfants de moins de 5 ans particulièrement à risque de développer une tuberculose lorsqu'ils sont contaminés ne sont pas ici représentés. La majorité des patients était de sexe masculin (61,2%).

**Tableau 1:** Caractéristiques de la population d’étude

Caractéristiques	Nombre	Pourcentage	Total
**Tranches d’âge**			
0-15 ans	0	0,0%	
16-20 ans	3	4,5%	
21-30 ans	20	29,9%	67(100%)
31-40 ans	19	28,4%	
41 -50 ans	11	16,4%	
50 ans et plus	14	20,9%	
**Sexe**			
Masculin	41	61,2%	67(100%)
Féminin	26	38,8%	
**Statut sérologique VIH**			
Négatifs	60	89,6%	
Positifs	7	10,4%	67(100%)

### Diagnostic de MTB par microscopie et PCR en temps réel

Le dépistage par bacilloscopie a révélé la présence de BAAR dans 59,3% (35/59) des prélèvements tandis que la recherche de mycobactéries par PCR en temps réel a donné 44,1% (26/59) de positivité. Le Tableau 2 montre les résultats du diagnostic de MTB par microscopie et par PCR en temps réel chez les patients venus au dépistage.

**Tableau 2:** Diagnostic de MTB par microscopie et par PCR en temps réel chez les patients dépistés

Diagnostic	Nombre	Pourcentage	Total
**Microscopie**			
Positives	35	59,3	59 (100,0)
Negatives	24	40,7	
**PCR**			
Positives	26	44,1	59 (100,0)
Négatives	33	55,9	

### Performance de la microscopie par rapport à la PCR en temps réel

La comparaison de la spécificité et de la sensibilité de la microscopie pour la recherche de BAAR a été faite par rapport à la PCR en temps réel. Sur les 74 échantillons de notre population d’étude, seules les personnes venues au laboratoire pour dépistage ont été prises en compte soit 59 patients. La microscopie avait une sensibilité de 68% et une spécificité de 91%. Le calcul de la sensibilité et de la spécificité a été fait tel que défini dans le Tableau 3.

**Tableau 3:** Performance des tests de microscopie des BAAR comparativement à la PCR

	PCR Positif	PCR négatif	Total
Microscopie Positives	VP= 24	FP= 11	VP + FP= 35
Microscopie Négative	FN = 2	VN= 22	FN + VN= 24

### Résistance aux antituberculeux

### Fréquence des mutations

Les résultats de l’étude des mutations se présentent comme suit: sur 48 échantillons soumis au test de résistance, 9 possédaient au moins une mutation qui confère une résistance à la rifampicine ou à l'isoniazide soit un taux de 18,75%.

#### Mutations qui confèrent une résistance à la rifampicine

Les mutations du gène *rpo*B conférant une résistance à la rifampicine ont été retrouvées dans 7 échantillons sur 9 soit de 77,8%. L'analyse des isolats a permis d'identifier les mutations sur les codons suivants 516, 531 et 533 avec les fréquences respectives suivantes 11,1%, 33,3% et 11,1%. La mutation la plus élevée a été observée sur le codon 531. Une mutation double dans le codon 531 (Ser→Trp + Ser→Leu) a également été observée. Deux isolats possédant des mutations du gène rpoB dont la localisation n'a pas été déterminée ont été observés (C + rpoB 22,22%) (Tableau 4). Mutations qui confèrent une résistance à l'isoniazide Huit échantillons sur neuf 8/9 (88,9%) ont présenté des mutations du gène inhA conférant une résistance à l'isoniazide dont 7 sur le gène *inh*A C209T de la région régulatrice et un dont le site de mutation n'a pas été détecté par notre kit. Aucune mutation sur la région promotrice *ahp*C du gène n'a été observée.

**Tableau 4:** Mutations du gène rpoB

Codon Muté	Mutation	Acide aminé changé	Fréquence	Types de résistances induits
516	GAC-GTC	Asp-Val	1(11,1%)	Faible niveau de résistance
531	TCG-TGG	Ser-Trp	2 (22,2%)	Haut niveau de résistance
531	TCG-TGG et TCG-TTG	Ser-Trp et Ser-Leu	1(11,1%)	Haut niveau de résistance
533	CTG-CCG	Leu-Pro	1(11,1%)	Faible niveau de résistance
Indéterminé (C+ rpoB)			2 (22,2%)	ND
Négatif			2 (22,2%)	Sensible

Les mutations du gène *kat*G conférant une résistance à l'isoniazide sont présentes dans 33,3% des échantillons. Le codon 315 était le seul codon incriminé avec une double mutation observée Ser-Thr et Ser-Asn sur le gène *kat*G (Tableau 5).

**Tableau 5:** Mutations du gène inhA et katG

Codon Muté	Mutation	Acide aminé changé	Fréquence	Types de résistances induits
*Inh*A				
C209T	C209T		7(77,8%)	Résistance à faible niveau
Site non précisé			1(11,1%)	ND
Négatif			1(11,1%)	Sensible
*kat*G				
315	AGC-ACA	Ser-Thr	2(22,2%)	Résistance à haut niveau
315	AGC-ACA et AGC-AAC	Ser-Thr et Ser-Asn	1(11,1%)	Résistance à très haut niveau
Négatif			6(66,7%)	Sensible

### Multi-Résistance (MR)

Des mutations à la fois du gène *rpo*B et *inh*A ou *kat*G conférant une multi-résistance (MR) ont été détectés dans 7 échantillons. Les mutations multiples rencontrées (44,4%) sont à la fois des substitutions sur le codon 531 du gène *rop*B (Ser-Trp (TCG-TGG) et sur le codon 209 du gène *inh*A (209T). Deux substitutions (22,2%) conférant une résistance à l'isoniazide situées sur le codon 315 du gène *kat*G Ser-Thr (AGC-ACA) et sur le codon 209 du gène *inh*A (209T) de *Mycobactérium tuberculosis* ont été observées.

Les substitutions doubles à l'intérieur du même gène étaient présentes dans deux cas: une substitution sur le codon 531 du gène *rop*B (Ser-Trp (TCG-TGG) /Ser-Leu (TCG-TTG), cette mutation concerne les allèles 1 et 2 du gène *rop*B et une substitution sur le codon 315 du gène *kat*G Ser-Thr (AGC-ACA) /Ser-Asn (AGC-AAC). Cette dernière concerne les allèles 12 et 15 du gène *kat*G.

## Discussion

Dans cette étude la présence de *M. tuberculosis* dans les échantillons a été recherchée par microscopie et par PCR en temps réel. Le bacille a été retrouvé dans 35 prélèvements (59,3%) par microscopie et la PCR en temps réel a permis d'identifier 26 échantillons positifs soit 44,1% des cas. Nos résultats sont similaires à ceux de Drouillon et al. qui ont trouvé une prévalence élevée de *M. tuberculosis* par microscopie (78,6%) comparativement à la PCR en temps réel (57,1%) [11]. Ceci s'expliquerait par le fait que les BAAR observés sur lames à l'examen direct ne correspondent pas tous à des mycobactéries appartenant au complexe *Mycobacterium tuberculosis*.

Les mutations responsables de la résistance à la rifampicine et à l'isoniazide ont été recherchées aussi bien aussi chez les patients que dans les souches de MTB issues de la culture. Les mutations suivantes du gène *rpo*B ont été retrouvées: codons 516 (11,11%), 531 (33,33%), et 533 (11,11%). Le codon 531 présente la fréquence la plus élevée. Ce codon a été associé à une résistance élevée à la rifampicine ainsi qu'une résistance croisée avec toutes les rifampicines. Les mêmes types de mutations ont été observés à Taïwan par Lin *et al*. mais à des proportions différentes: 516 (4,5%), 531 (68,2%), 533(9,1%) avec toujours une prédominance des mutations du codon 531 [12]. La présence des mutations sur le codon 531 du gène *rpo*B pourrait expliquer l’échec de traitement chez certains de nos patients aux médicaments de première ligne dont la rifampicine. Nos résultats sont différents de ceux de Miotto et al. au Burkina Faso en 2009 qui ont trouvé une prédominance du codon 526 (31,2%) contre 9,4% pour le codon 531 du gène *rpo*B [13].

Egalement, les résultats obtenus dans cette étude sont différents de ceux rapportés par Sheng et al. en Chine Taiwan, qui ont trouvé une prédominance de la mutation du codon 526 (73,2%) et une faible proportion des mutations du codon 531 (3,5%) [14]. En effet, la prévalence du codon 531 varie selon les régions géographiques [14, 15].

Dans cette étude nous avons identifié une grande proportion (88,8%) de mutations C209T au niveau de la région régulatrice du gène de l’*inh*A. En effet, les mutations se situant dans la région régulatrice, sont connues pour causer une surexpression du gène *inh*A renforçant l'expression de la résistance de *M. tuberculosis* à l'isoniazide [16]. Les mutations dans l’*inh*A ou dans sa région régulatrice sont habituellement associées avec une résistance de faible niveau et sont moins fréquentes que les mutations du gène *kat*G. Dans les souches de *M. tuberculosis* résistantes à l'isoniazide et présentant des mutations *inh*A, on pourrait trouver des mutations supplémentaires de *kat*G qui conféreraient des niveaux plus élevés de résistance à l'isoniazide. La mutation *kat*G a été retrouvée chez 33,33% des souches de *M. tuberculosis* dans cette étude Nos résultats sont similaires à ceux rapportés en Espagne par Torres et al et ceux de Telenti et al, qui ont trouvé respectivement des prévalences de 68,1% et 46% de la mutation C209T [15, 16]. Ainsi, environ 70 à 80% des résistances cliniques à l'INH peuvent être attribuées aux mutations sur les gènes *kat*G et *inh*A [17].

Nous avons observé singulièrement des mutations au niveau du codon 315 de *kat*G avec une double mutation Sr→Thr et Sr→Asp. La mutation S315T dans *kat*G est la mutation la plus courante dans les souches résistantes à l'isoniazide, et se rencontre dans 50% à 95% des isolats cliniques [18, 19]. L'existence de cette mutation expliquerait les échecs de traitement de première ligne chez les patients. La mutation du codon 315 de *kat*G a déjà été associée à la résistance à l'isoniazide dans deux précédentes études mais ces études précédentes n'ont pas trouvé comme dans le cas présent une double mutation sur le codon 315 [20, 21].

Les mutations dans les positions 531 et 516 du gène *rpo*B que nous avons trouvées sont parmi les mutations les plus fréquemment observées dans les souches résistantes à la rifampicine. Les mutations dans *rpo*B entraînent généralement une résistance de haut niveau ainsi qu'une résistance croisée avec toutes les rifampicines [18]. Les mutations dans *inh*A ne provoquent pas seulement la résistance à l'isoniazide, mais elles confèreraient également une résistance croisée à l’égard d'un médicament structurellement proche, tel que l’éthionamide [22]. Nous avons identifié également une multi résistance de MTB chez 10,44% des patients (7/67), c´est-à-dire des patients ayant présenté à la fois des mutations sur le gène *rop*B et sur le gène *inh*A ou *kat*G. Ce taux est similaire par rapport à ceux rapportés par Koeck à Djibouti en 2002 qui a trouvé un taux de (11%) [23], tandis la prévelance rapportée par cette étude est supérieure à celle de Tessema et al en Éthiopie en 2009 et par Torres et al en Espagne en 2002 qui ont trouvé respectivement des prévalences de 5% et (2,3%) d'isolats de *M. tuberculosis* multi résistants [15, 24].

## Conclusion

Dans cette étude, les mutations des gènes *rpo*B, *kat*G et *inh*A associées à une résistance à la rifampicine et à l'isoniazide ont été observées. Les causes qui induisent ces multirésistances au Burkina Faso seraient la mauvaise observance des traitements par les patients et la prescription systématique de la rifampicine et de l'isoniazide sans la recherche préalable de résistances. Le test MTB Real-TM Résistance 8 est bien approprié pour le diagnostic des résistances à l'isoniazide et la rifampicine. Il présente l'avantage d'avoir un temps de réalisation très court et donne la possibilité d'utiliser un grand nombre d’échantillons à la fois. Malheureusement le test moléculaire par PCR en temps réel coût très cher et en plus, il faudra des techniciens bien formés et un plateau technique adéquat pour effectuer ces types d'analyses. Sa mise en oeuvre et sa vulgarisation permettra de réduire le temps d'attente avant la mise sous traitement. Mais il devrait être associé à la culture pour mieux déterminer les résistances primaires et secondaires des souches aux antituberculeux.
