# Corrigendum: Diagnostic moléculaire du complexe Mycobacterium tuberculosis résistant à l’isoniazide et à la rifampicine au Burkina Faso

**DOI:** 10.11604/pamj.2018.30.284.15545

**Published:** 2018-08-23

**Authors:** Désire Ilboudo, Cyrille Bisseye, Florencia Wendkuuni Djigma, Souba Diande, Albert Théophane Yonli, Jean Telesphore Valerie Bazie, Rebeca Tégwindé Compaore, Charlemagne Gnoula, Tamboura Djibril, Rémy Moret, Virginio Pietra, Damintoti Simplice Karou, Martial Ouedraogo, Jacques Simpore

**Affiliations:** 1 Centre de Recherche Biomoléculaire Pietro Annigoni, CERBA/LABIOGENE, Université de Ouagadougou, Ouagadougou, Burkina Faso; 2 Laboratoire National de Référence de Mycobactéries, Programme National Tuberculose, Ouagadougou, Burkina Faso; 3 Centre Médical St Camille, Ouagadougou, Burkina Faso; 4 Ecole Supérieure des Techniques Biologiques et Alimentaires (ESTBA-UL), Université de Lomé, Lomé, Togo; 5 Unité de Formation et de Recherche en Sciences de la Santé (UFR/SDD), Université de Ouagadougou, Ouagadougou, Burkina Faso

**Keywords:** Mycobacterium tuberculosis, résistance, rifampicine, isoniazide

## Abstract

Ce corrigendum modifie l'article original: Diagnostic moléculaire du complexe Mycobacterium tuberculosis résistant à l’isoniazide et à la rifampicine au Burkina Faso. The Pan African Medical Journal. 2015;21:73. doi:10.11604/pamj.2015.21.73.5494.

## Corrigendum

La version originale [1] de cet article contient les noms suivants: Ilboudo Désire, Bisseye Cyrille, Djigma Florencia, Diande Souba, Yonli Albert, Bazie Jean Telesphore Valerie, Compaore Rebecca, Gnoula Charlemagne, Tamboura Djibril, Moret Rémy, Pietra Virginio, Karou Damintoti Simplice, Ouedraogo Martial, Simpore Jacques au lieu de Désire Ilboudo, Cyrille Bisseye, Florencia Djigma, Souba Diande, Albert Yonli, Jean Telesphore Valerie Bazie, Rebecca Compaore, Charlemagne Gnoula, Djibril Tamboura, Rémy Moret, Virginio Pietra, Damintoti Simplice Karou, Martial Ouedraogo, Jacques Simpore.
